# Relation of Transversus Abdominis to Rectus Abdominis Muscle in Various Anatomical Landmark Levels: A Cadaveric Study

**DOI:** 10.1055/a-2350-8420

**Published:** 2024-08-06

**Authors:** Totsapol Surichamorn, Thiti Tantitham

**Affiliations:** 1 Division of Plastic and Maxillofacial Surgery, Department of Surgery, Ramathibodi Hospital, Mahidol University, Bangkok, Thailand

**Keywords:** transversus abdominis, rectus abdominis, posterior component separation

## Abstract

**Background** Posterior Component Separation (PCS) is a surgical technique used in abdominal wall reconstruction. Understanding the relationship between the rectus abdominis and transversus abdominis muscles and the location of intercostal nerves is crucial for minimizing nerve injury during PCS. This cadaveric study aimed to investigate these anatomical relationships and propose practical guidelines for safer PCS procedures.

**Methods** Eighteen fresh cadavers were dissected to assess the overlap or separation of the rectus abdominis and transversus abdominis muscles at seven abdominal levels. The distance of intercostal nerves from the lateral border of the rectus abdominis was measured.

**Results** The study found that the muscles overlapped at the xiphoid and upper abdominal levels but began to separate below the 2/4 upper to umbilicus level. Intercostal nerves entered at varying distances from the lateral edge of the rectus abdominis, suggesting that levels above the 3/4 upper to umbilicus level are relatively safe for dissection.

**Conclusion** The study recommends initiating the first incision for PCS between the subxiphoid and 2/4 upper to the umbilicus, based on observed muscle relationships and nerve distances. This practical approach enhances safety and simplifies decision-making during surgery.

## Introduction

Abdominal wall defects are common issues encountered in the fields of general surgery and plastic surgery. The primary objective of treatment is to restore abdominal wall integrity, which can be broadly categorized into two approaches: mesh-based repair and tissue-based repair. In tissue-based repair, a myofascial flap is advanced to cover the defect, or the Component Separation Technique (CST) is employed. The latter, based on the principle of releasing tension over the lateral muscles, facilitates the medial advancement of the rectus abdominis, followed by suturing without tension.

The first CST described was the Anterior Component Separation (ACS). Subsequently, Ramirez et al 1 introduced Posterior Component Separation (PCS) in 1990 by adapting the abdominal wall repair technique by Rives–Stoppa. While the ACS releases the external oblique aponeurosis, Ramirez et al's technique focuses on releasing the transversus abdominis aponeurosis.

In 2012, Novitsky et al 2 and Krpata et al 3 conducted a study comparing the advantages, disadvantages, and safety of the ACS. The study concluded that the PCS provides equivalent myofascial advancement with significantly lower wound morbidity when compared to the ACS. Moreover, it emphasized the importance of avoiding injury to the intercostal nerve, which travels between the internal oblique and transversus abdominis muscles. Therefore, when performing the PCS dissection, it is advisable to initiate the procedure at the midline celiotomy, entering the posterior rectus sheath 1 cm laterally from the midline. Subsequently, the dissection of the transversalis fascia should be performed before encountering the intercostal nerve. This dissection should progress from the cephalic to the caudal direction in the area of overlap between the transversus abdominis and rectus abdominis muscles, which is considered safe for identifying the intercostal nerve compared to the area below the umbilical level.

Thus, concerning the development of PCS, knowing the exact point of overlap between the transversus abdominis and rectus abdominis muscles can expedite celiotomy incision and the identification of the intercostal nerve.

Despite an extensive review of the literature, including sources such as Grey's Anatomy, 4 Netter's Atlas of Human Anatomy, 5 Netter's Clinical Anatomy, 6 Grant's Atlas of Anatomy, 7 Snell's Clinical Anatomy, 8 Bailey & Love's Essential Clinical Anatomy, 9 and Last's Anatomy, 10 there is a lack of clear clarification regarding the relationship between the rectus abdominis and transversus abdominis muscles. They depict either no overlap between the two muscles, or lack of distinct level of the overlapped segment.

In 2018, Punekar et al 11 conducted a study titled “Redefining the Rectus Sheath: Implications for Abdominal Wall Repair.” In this study, CT scan imaging was utilized to identify the relationship between the rectus abdominis muscle and transversus abdominis in various anatomical positions based on spinous level. The study discovered that, at the costal margin plane (T12–L1, 4.2 cm), all subjects exhibited a significant presence of the transversus abdominis within the rectus sheath (the overlap between the rectus abdominis and transversus abdominis muscles). Furthermore, 99% had transversus abdominis presence within the rectus sheath at L1 to L2 (3.2 cm), 86% at the level of the 12th rib (L2–L3, 1.4 cm), 36% at the umbilicus (L3–L4), and 2% slightly above the posterosuperior iliac spine (L5–S1). These findings have practical implications for patient selection and surgical technique.

However, in terms of practical use, we believe that using the anterior abdominal surface anatomy as a landmark in the prone position, the standard position for performing PCS, rather than the spinous level, would be more efficient in the operating room. Furthermore, we propose the need for an anatomical dissection study.

## Methods

This study received approval from the Ethics Committee of Mahidol University, and the cadavers used in this study were sourced from leftover cadavers from the Clinical Anatomy and Research Education Laboratory Project workshop. A total of 20 cadavers were included in the study. However, 2 cadavers were excluded due to previous abdominal wall dissections, leaving 18 cadavers for the study.

**Fig. 1:**
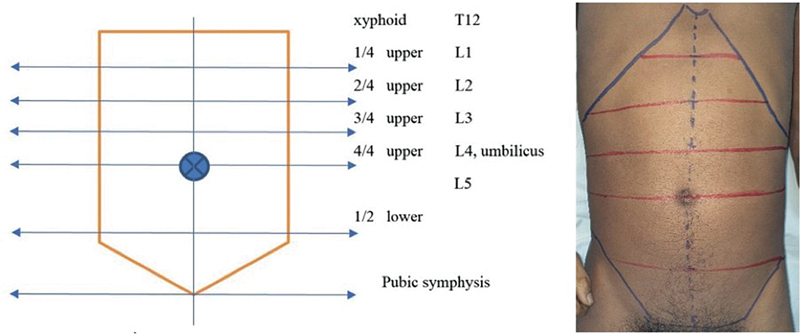
Description of markings at various abdominal levels.

The dissection process began with the cadavers in the supine position. The abdominal wall was marked at various levels, including the xiphoid, T12, 1/4 upper umbilicus, 2/4 upper umbilicus, 3/4 upper umbilicus, 4/4 or umbilicus level, 1/2 lower to the umbilicus, 2/2 lower to the umbilicus, or pubic symphysis level, to simplify the marking process during surgery ( Fig. 1 ).

**Fig. 2:**
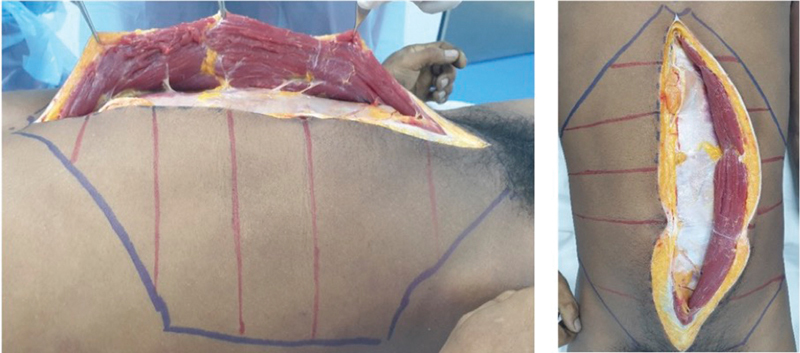
This figure shows the left posterior rectus sheath from the xiphoid to the pubic symphysis level. Intercostal nerves are shown underneath the rectus muscle.

Subsequently, a midline laparotomy was performed from the subxiphoid level to the pubic symphysis level using a number 15 blade. The space between the posterior rectus muscle and the posterior rectus sheath was accessed by dissecting 1 cm laterally to the linea alba, allowing the identification of the transversus abdominis and the transversus fascia. The dissection began on the left side of the cadaver ( Fig. 2 ). All intercostal nerves were identified to the extent possible, and the distance of the nerve from the lateral border of the rectus abdominis was recorded in millimeters.

**Fig. 3:**
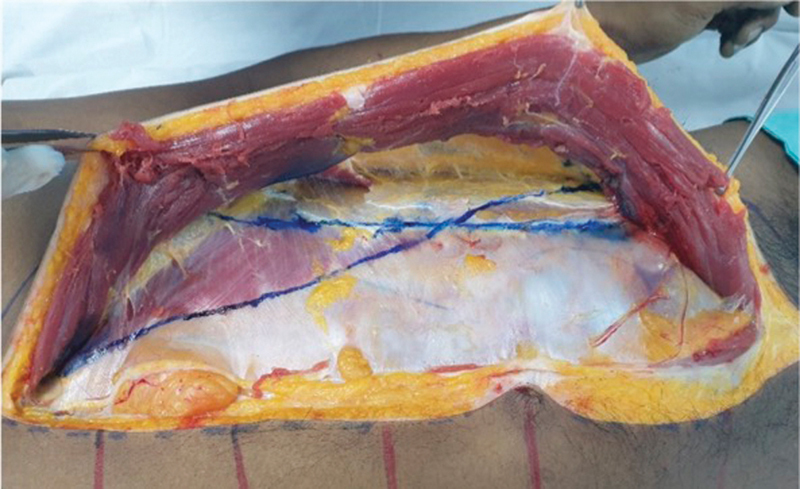
Identification of the lateral border of rectus abdominis and medial border of transversus abdominis in blue ink.

After all relevant structures were fully dissected, the edge of the rectus abdominis muscle was marked in blue ink on the rectus sheath, while the edge of the transversus abdominis muscle was marked in blue ink as well ( Fig. 3 ).

**Fig. 4:**
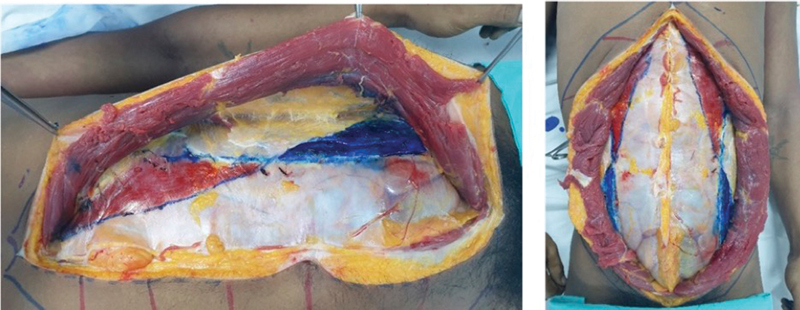
Left; the red area represents overlapping area between rectus abdominis and transversus abdominis muscle. The blue area represents the separating area between rectus abdominis and transversus abdominis muscle. The right-hand side figure shows both sides of the cadaver.

The overlapping area between the transversus abdominis and rectus abdominis muscles was marked in red ink ( Fig. 4 , left), while the separating area between the two muscles was marked in blue ink ( Fig. 4 , right). This marking procedure was performed on both sides of the cadaver.

The relationship between the rectus abdominis and transversus abdominis muscles at various levels was recorded in terms of negative values if the two muscles overlapped and positive values if the two muscles were separated. Once the dissection on the left side was completed, the same procedure was replicated on the right side of the cadaver ( Fig. 4 , right). The data, measured in millimeters for the intercostal nerve, were recorded separately for the right and left sides in terms of the number of nerves found and the distance of the nerve measured from the lateral border of the rectus abdominis at each level. Nerve data were recorded in millimeters, and all data were collected for statistical analysis.

### Statistical Analysis

All cadaver characteristics and data related to overlap or separation distances at various levels were analyzed using mean, median, maximum, and minimum values.

## Results

**Fig. 5:**
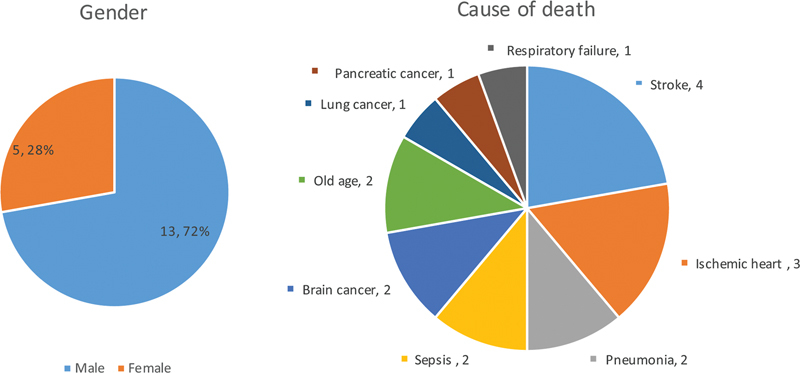
Demographic data; gender and cause of death. TA, transversus abdominis.

Eighteen fresh cadavers were enrolled in the study, with two cadavers excluded due to not meeting the inclusion criteria. The demographic data revealed that the majority of the cadavers were male (72.22%), with the remaining cadavers being female (27.78%). The average age was 73.16 ± 9.61 years (maximum 89 years, minimum 56 years). Various causes of death were observed, including hemorrhagic stroke in three cadavers, as well as pneumonia, brain cancer, ischemic heart disease, sepsis, lung cancer, pancreatic cancer, and respiratory failure, with none of them having previous abdominal wall injuries. A detailed breakdown of all demographic data is provided ( Fig. 5 ).

Regarding the relationship between the transversus abdominis and the rectus abdominis muscle at various surface anatomy levels, the data showed that at the xiphoid level, the transversus abdominis overlapped with the rectus abdominis with a mean overlap of 42.00 mm. At the 1/4 upper to umbilicus level, the transversus abdominis overlapped with the rectus abdominis with a mean overlap value of 32.25. At the 2/4 upper to umbilical level, the transversus abdominis overlapped with the rectus abdominis with a mean overlap value of 21.25. At the 3/4 upper to umbilical level, the transversus abdominis overlapped with the rectus abdominis with a mean value of 12.22 mm. At the level of 4/4 or the umbilical level, the transversus abdominis separated from the rectus abdominis, with a mean value of 3.61 mm for the separation distance. At the 1/2 lower to the umbilicus level, the transversus abdominis separated from the rectus abdominis, with a mean value of 21.38 mm for the separation distance. Finally, at the 2/2 lower to the umbilicus or pubic symphysis level, the transversus abdominis separated from the rectus abdominis, with a mean value of 45.72 mm for the separation distance ( Table 1 ).

**Table 1:** Mean distance of transversus abdominis and rectus abdominis at various location

	Mean TA distance on right side (mm) (SD)	Mean TA distance on left side (mm) (SD)	Mean TA distance on both sides (mm) (SD)
Xyphoid	− 41.94 (7.53)	− 42.05 (7.86)	− 42.00 (7.58)
1/4 upper to umbilicus	− 32.66 (8.80)	− 31.83 (9.56)	− 32.25 (9.07)
2/4 upper to umbilicus	− 21.94 (10.82)	− 20.55 (9.69)	− 21.25 (10.15)
3/4 upper to umbilicus	− 12.88 (10.96)	− 11.55 (9.58)	− 12.22 (10.17)
4/4 (umbilicus)	3.55 (7.99)	3.66 (7.76)	3.61 (7.76)
1/2 lower to umbilicus	22.11 (8.14)	20.66 (7.99)	21.38 (7.98)
2/2 lower to umbilicus	45.33 (8.00)	46.11 (7.58)	45.72 (7.69)

Abbreviations: TA, transversus abdominis; SD, standard deviation.

**Fig. 6:**
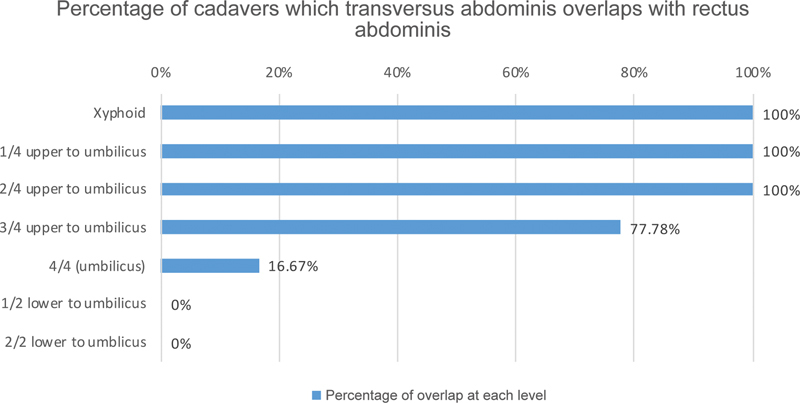
Percentage of cadavers whose transversus abdominis and rectus abdominis overlapped each other at each abdominal level.

At the xiphoid level, the transversus abdominis overlapped with the rectus abdominis in all cadavers (100%, 18/18), while at the 1/4 upper to umbilicus level, this overlap was observed in all cadavers (100%, 18/18). At the 2/4 upper to umbilical level, the transversus abdominis also overlapped with the rectus abdominis in all cadavers (100%, 18/18). However, at the 3/4 upper to umbilical level, the transversus abdominis began to separate from the rectus abdominis, overlapping in 14 cadavers out of 18 (77.78%). Thus, in 4 of the 18 cadavers, both muscles separated from each other (22.23%). At the level of 4/4 or the umbilical level, the transversus abdominis separated from the rectus abdominis in 15 cadavers out of 18 (83.33%), while 3 cadavers out of 18 still had both muscles overlapping (16.67%). Below this level, at 1/2 lower to the umbilicus, the transversus abdominis separated from the rectus abdominis in all cadavers (100%, 18/18), as did at the 2/2 lower to the umbilicus or pubic symphysis level (100%, 18/18; Fig. 6 ).

**Fig. 7:**
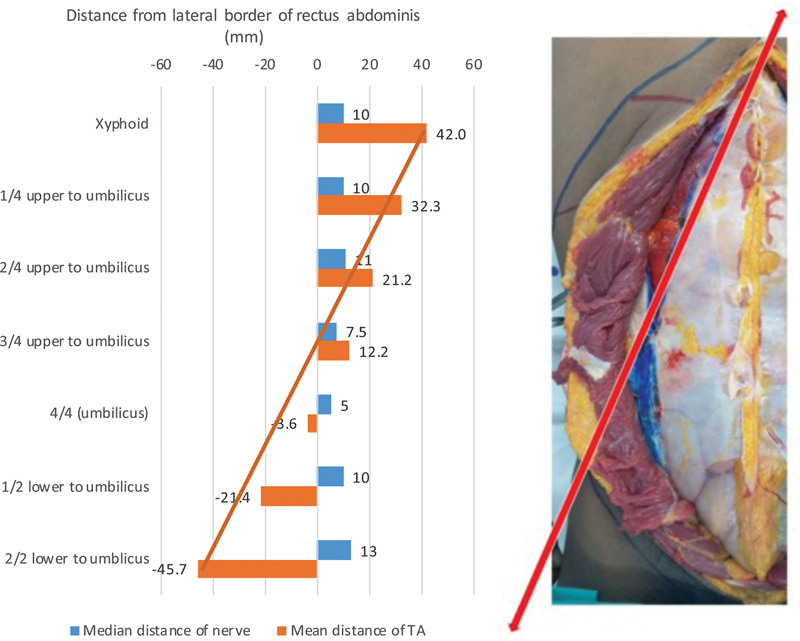
Description of the relationship of distance from lateral border of rectus abdominis muscle to transversus abdominis muscle, and intercostal nerve in various levels. TA, transversus abdominis.

**Fig. 8:**
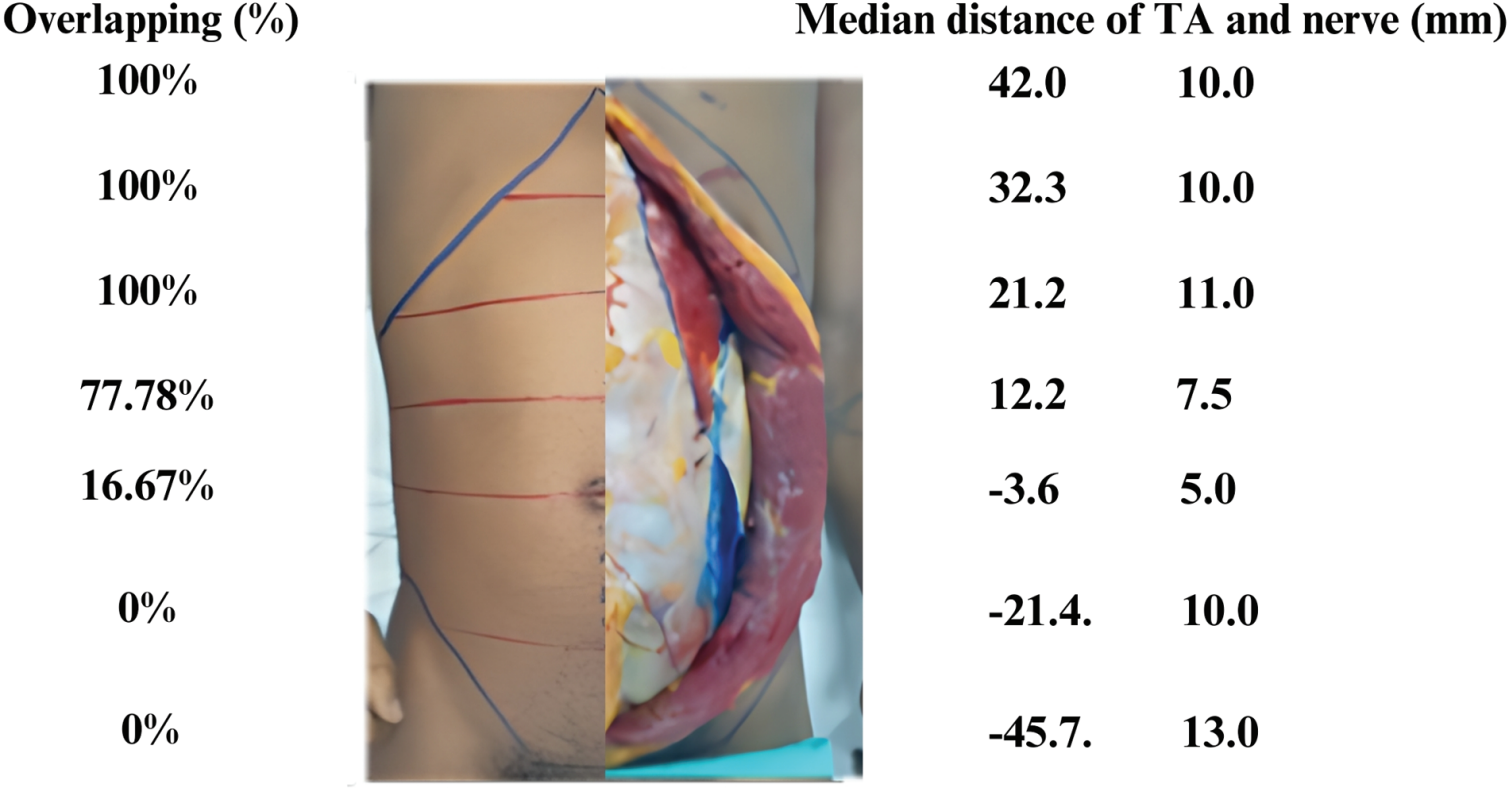
Summary of important findings including percentage of muscle overlapping, median distance from lateral border of rectus abdominis muscle to transversus abdominis muscle, and intercostal nerve in various levels. TA, transversus abdominis.

Regarding nerve data, 16 out of 18 cadavers were dissected for nerve location and distance, with 2 cadavers not receiving dissection due to technical difficulties. The distance of the nerve was measured from the lateral border of the rectus abdominis muscle on each side. We reported the finding as median distance due to the skewed distribution of data. At the xiphoid level, the nerve was not found on the right side, and on the left side, one nerve was found in 1 body out of 16 bodies (6.25%). The median nerve distance was 10 mm from the lateral edge of the rectus abdominis. At the 1/4 upper to umbilicus level, one nerve was found in 10 bodies out of 16 (62.50%) on the right side, while on the left side, one nerve was found in 8 bodies out of 16 (50%). The median nerve distance was 10 mm from the lateral edge of the rectus abdominis. At the 2/4 upper to umbilicus level, one nerve was found in 9 bodies out of 16 (56.25%) on the right side, while on the left side, one nerve was found in 12 bodies out of 16 (75%). The median nerve distance was 11 mm from the lateral edge of the rectus abdominis. At the 3/4 upper to umbilicus level, one nerve was found in 13 bodies out of 16 (81.25%) on the right side, while on the left side, one nerve was found in 15 bodies out of 16 (93.75%). The median nerve distance was 7.5 mm from the lateral edge of the rectus abdominis. At the 4/4 or umbilical level, one nerve was found in 11 bodies out of 16 (64.71%) on the right side, while on the left side, one nerve was found in 9 bodies out of 16 (56.25%). The median nerve distance was 5 mm from the lateral edge of the rectus abdominis. At the 1/2 lower to the umbilicus level, one nerve was found in 3 bodies out of 16 (18.75%) on the right side, while on the left side, one nerve was found in 5 bodies out of 16 (31.25%). The median nerve distance was 10 mm from the lateral edge of the rectus abdominis. At the 2/2 lower to the umbilicus or pubic symphysis level, one nerve was found in 1 body out of 16 (6.25%) on the right side, but no nerve was found on the left side. The median nerve distance was 13 mm from the lateral edge of the rectus abdominis ( Table 2 ; Figs. 7 and 8 ).

**Table 2:** Description of percentage of nerves found and distance of nerve from lateral edge of rectus abdominis at each level

Level	Median distance of both sides (mm; p25, p75)	Right side		Left side	
		Percentage of nerves found	Median distance (mm; p25, p75)	Percentage of nerves found	Median distance (mm; p25, p75)
Xyphoid	10	0	0	6.25%	10
1/4 upper to umbilicus	10 (10, 13)	62.50%	11 (10, 13)	50.00%	10 (10, 12.5)
2/4 upper to umbilicus	11 (4, 15)	56.62%	11 (5, 15)	75.00%	10.5 (4, 15)
3/4 upper to umbilicus	7.5 (3.5, 13)	81.25%	7 (4, 10)	93.75%	10 (3, 13)
4/4 (umbilicus)	5 (2, 10)	64.71%	5 (2, 10)	56.25%	5 (2, 10)
1/2 lower to umbilicus	10 (10, 14.5)	18.75%	14 (10, 15)	31.25%	10 (10, 10)
2/2 lower to umbilicus	13	6.25%	13	0	0

## Discussion

In abdominal wall reconstruction, the midline defect sparing both rectus muscles, such as incisional hernia, can be repaired with the PCS technique. Careful separation between muscle layers while preserving the intercostal nerve is critical for a successful reconstruction. Many previous studies have suggested that the area where both the transversus abdominis muscle and rectus abdominis muscle overlap is the most appropriate area for identifying the nerve, as opposed to areas where the muscles are separated, for safety reasons. 2 12 13 14 15 16 17 This aligns with the findings of the study by Punekar et al (2018) titled “Redefining the Rectus Sheath: Implications for Abdominal Wall Repair” 11 which used CT scans to locate the relationship between the transversus abdominis muscle and the rectus abdominis muscle in terms of separation or overlap at various abdominal levels, as marked by spinous level. Their results indicated a significant presence of the transversus abdominis within the rectus sheath at the costal margin plane (T12–L1, 4.2 cm), L1 to L2 (3.2 cm), L2 to L3 (1.4 cm), and even at the umbilicus level (L3–L4) to some extent, with only 2% showing presence slightly above the posterosuperior iliac spine (L5–S1).

However, the current study suggests a simpler and more practical approach. Rather than relying on spinous level markings, the authors propose using surface anatomy markings in the supine position, which is the routine position for performing PCS. This approach aims to streamline the decision-making process in the operating room. The study also adds valuable insights regarding the specific levels at which the transversus abdominis and rectus abdominis muscles overlap or separate.

The data showed that, from the xiphoid level to 2/4 upper to the umbilicus level, the two muscles overlapped in 100% of the cadavers. Below this level, the transversus abdominis muscle began to separate from the rectus abdominis muscle. At the 3/4 upper to umbilical level, both muscles overlapped in 77.78% of cases, with a mean overlap distance of 12.22 mm. However, intercostal nerves entered at a median distance of 7.5 mm, suggesting that this level may still be relatively safe for dissection.

The study concludes by recommending that surgeons performing PCS consider initiating the first incision at the level from the subxiphoid to 2/4 upper to the umbilicus. This level was chosen based on the observed relationship between the two muscles and their relative distances to the intercostal nerves. In midline abdominal defects, as long as the anatomy and the integrity of both rectus abdominis and transversus abdominis are kept intact, this approach will not only enhance safety by reducing the risk of accidental nerve injury but also streamline the decision-making process during surgery.

The study's findings align with the previous study by Punekar et al (2018), 11 even though the methods used were different. The practicality and efficiency of using surface anatomy markings in the supine position could provide a valuable alternative to the spinous level-based approach.

### Conclusion

The study has clarified the relationship between the rectus abdominis and transversus abdominis muscles, and the location of intercostal nerves at various anatomical levels. It proposes a simple and efficient method for avoiding intercostal nerve injury during PCS procedures by recommending the initiation of the first incision at the level from the subxiphoid to 2/4 upper to the umbilicus.

### Limitations

The study acknowledges several limitations. The sample size was relatively small due to the limited availability of fresh cadavers, and the majority of the cadavers were older individuals, potentially limiting the generalizability of the findings. Additionally, the dissections were performed with the aid of headlight illumination and a single assistant, without the use of loupe magnification, which may have impacted the identification of nerves. Future studies should aim for larger and more diverse samples, as well as improved dissection procedures and instrumentation.
